# Species-Level Analysis of Human Gut Microbiota With Metataxonomics

**DOI:** 10.3389/fmicb.2020.02029

**Published:** 2020-08-26

**Authors:** Jing Yang, Ji Pu, Shan Lu, Xiangning Bai, Yangfeng Wu, Dong Jin, Yanpeng Cheng, Gui Zhang, Wentao Zhu, Xuelian Luo, Ramon Rosselló-Móra, Jianguo Xu

**Affiliations:** ^1^State Key Laboratory of Infectious Disease Prevention and Control, Chinese Center for Disease Control and Prevention, National Institute for Communicable Disease Control and Prevention, Beijing, China; ^2^Shanghai Public Health Clinical Center, Shanghai Institute for Emerging and Re-emerging Infectious Diseases, Shanghai, China; ^3^Research Units of Discovery of Unknown Bacteria and Function, Chinese Academy of Medical Sciences, Beijing, China; ^4^Peking University Clinical Research Institute, Beijing, China; ^5^Marine Microbiology Group, Department of Ecology and Marine Resources, Instituto Mediterráneo de Estudios Avanzados (IMEDEA), Esporles, Spain; ^6^Institute of Public Health, Nankai University, Tianjing, China

**Keywords:** metataxonomics, species-level phylotypes, resident bacteria, gut microbiota, potential pathogenic species

## Abstract

The current understanding of human gut microbial community is mainly limited to taxonomic features at the genus level. Here, we examined the human gut microbial community at the species level by metataxonomics. To achieve this purpose, a high-throughput approach involving operational phylogenetic unit analysis of the near full-length 16S ribosomal RNA (rRNA) gene sequence was used. A total of 1,235 species-level phylotypes (SLPs) were classified in the feces of 120 Chinese healthy individuals, including 461 previously classified species, 358 potentially new species, and 416 potentially new taxa, which were categorized into low, medium, and high prevalent bacteria groups based on their prevalence. Each individual harbored 186 ± 51 SLPs on average. There was no universal bacterial species shared by all the individuals. However, 90 ± 19 of 116 SLPs were shared in the high prevalent bacteria group. Thirty-two out of thirty-eight species in the high prevalent bacteria group detected in this study were also found in at least one previous study on human gut microbiota based on either culture-dependent or culture-independent approaches. Through compositional analysis, a hierarchical clustering of the prevalence and relative abundance of the 1,235 SLPs revealed two types of gut microbial communities, which were dominated by *Prevotella copri* and *Bacteroides vulgatus*, respectively. The type dominated by *P. copri* was more prevalent in northern China, while the *B*. *vulgatus-*dominant type was more prevalent in southern China. Therefore, P- and B-type gut microbial communities in China were proposed. It was found that 166 out of 461 known bacterial species have been previously reported as potential pathogens, and the individuals sampled for this study harbored 20 of these potential pathogenic species on average. The top two most abundant and prevalent potential pathogenic species were *Klebsiella pneumoniae* and *Bacteroides fragilis*.

## Introduction

The first step to understand the relationship between gut microbes and their hosts is to thoroughly characterize the microbiota in healthy individuals (Lozupone et al., 2012). This is important because its compositional characterization can enhance microbiota-based diagnostics and therapies and even prevent various diseases (Costea et al., 2018). The analysis of 16S ribosomal RNA (rRNA) gene amplicons using next-generation sequencing platforms has completely revolutionized the culture-independent research study on microbial diversity. However, the general short-length 16S rRNA sequences usually limit the taxonomic classification of the microbial community to the genus level (Yarza et al., 2014). Thus, the reliable and accurate identification of hierarchies within taxonomic system requires full-length 16S rRNA sequences. Here, we investigated the composition of gut microbial community in 120 healthy Chinese individuals. To achieve this, a recently developed method, metataxonomics, which characterized the gut microbial community of vulture and Tibetan antelope at the species level, was employed (Meng et al., 2017; Bai et al., 2018), using near full-length 16S rRNA sequences obtained from a Pacific Biosciences (PacBio) single-molecule real-time (SMRT) sequencing platform, which provided long-read sequences (Rosselló-Móra and Amann, 2015). Thereafter, a phylogenetic inference approach was conducted, which greatly increased the precision of characterization at the species level, thereby allowing us to reconstruct a *de novo* tree by phylogenetic filters. It markedly diminished the influence of sequence errors and indels (Mora-Ruiz et al., 2016) and increased the reliability and accuracy of our analysis (Yarza et al., 2008; Meng et al., 2017). In this study, a total of 120 Chinese human gut microbiota were analyzed by metataxonomics, and 1,235 SLPs (species-level phylotypes) were detected. Each Chinese individual had a personalized microbiota with a unique taxonomic composition, harboring 90 ± 19 of the 116 SLPs in the high prevalent bacteria group.

## Materials and Methods

### Study Design and Sampling

Human individuals in seven geographically historical regions of China (one province in each region), covering the main territory, such as northeast, northwest, center, east, southeast, southwest, and south, were targeted for sample collection (Supplementary Figure 1). Around 200 healthy individuals from each sampling site were initially recruited by the scientists in the local Center for Disease Control and Prevention (CDC) for further screening the qualified participants. The individuals with clinical or subclinical diseases, alcohol addictive behaviors, or having any habit that may influence the gut microbiota composition were excluded. The exclusion criteria included (1) individuals with digestive tract symptoms (loss of appetite, nausea, vomiting, diarrhea, constipation, abdominal pain, etc.); (2) individuals with digestive tract diseases (gastric and duodenal ulcer, gastroenteritis, gastrointestinal infections, intestinal obstruction, intestinal dysbacteriosis, gastrointestinal bleeding, gastrointestinal dysfunction, chronic diarrhea, intestinal parasites, and other diseases that may relate with gastrointestinal functions); (3) individuals with severe systemic diseases (chronic obstructive pulmonary disease, diabetes, metabolic syndrome, tumor, endocrine disorder, autoimmune diseases, urinary system diseases, HIV infection, anemia, etc.); (4) individuals with cardiovascular diseases (hypertension, coronary heart disease, stroke, etc.); (5) individuals with a history of uncontrolled epilepsy, central nervous system diseases, or mental disorders; (6) alcohol- or drug-dependent individuals that participated in a drug intervention or under certain medical treatments; (7) individuals taking antibiotics in the latest half year; (8) individuals with heavy drinking habit (alcohol, coffee, or functional drinks, e.g., exceeding 250 ml Chinese spirits/time, or three cups/a day, or four cans red bull energy drink/day, respectively); and (9) individuals that were pregnant and breastfeeding.

To better represent the provincialism, only the individuals who had resided in the sampling region for at least 6 months during the last 12 months were included. To better understand the local diversity, only one individual from each family was included. Considering the variation in food sources, an equal number of residents in town and rural village was included. The Body Mass Index of the individuals was in the range from 18 to 27. The overweight or underweight individuals were not included. The individuals who fulfilled the criteria of this study were further selected by our research team members in the CDC of China at Beijing. The individuals who were willing to participate in the study were informed about the study plan and asked to sign the written informed consent. Then, a standard questionnaire was performed to collect demographic information, anthropometric measurements, cognitive and health status, and clinical anamnesis. The medical examination for all qualified participants was conducted by local doctors, which included basic physical measurement, routine blood test, and examination for blood glucose, blood lipid, liver function, and renal function.

Sampling was performed between September and December 2016. Approximately 50 g feces was collected from each individual into sterile tubes and placed in an ice box for immediate transportation into the in laboratory of local CDC. Each sample was subsequently processed and distributed into three tubes, stored in deep freezer at −20°C with Uninterruptible Power System, and transported to our laboratory in Beijing, where all the experiments were further performed. To eliminate the possible impact of sample transportation, at least one DNA sample was extracted from each individual for 16S rRNA gene sequencing by our team members at the local CDC laboratory and transported our laboratory as well at Beijing. A total of 155 healthy individuals were selected from the seven sampling sites in this study, with 22 excluded after clinical examination and laboratory tests, due to hypertension or high serum concentration level. DNA samples from the rest of the individuals were processed and sequenced, among which 13 samples were further excluded due to low-yield PCR products or low read numbers generated (*N* < 3000).

### Full-Length 16S rDNA Amplification and Sequencing

DNA was extracted from human fecal samples (aliquots ranging between 150 and 200 mg) in the local laboratory of CDC. In order to get a better yield, the stool sample went through one step of mechanical violent oscillation. Then, general DNA extraction was operated with the QIAamp Fast DNA Stool Mini Kit (Qiagen, cat. 51604) according to the manufacturer’s instructions. After all the DNA samples were transported to our laboratory, amplification of 16S rRNA genes was conducted using the universal primer set 27F/1492R (5′-AGAGTTTGATCCTGGCTCAG-3′) and 1492R (5′-GNTACCTTGTTACGACTT-3′) with 16 nt symmetric (reverse complement) barcodes tagged at the 5′ end, which were designed for PacBio system allowing multiplex samples in a single cell and run. PCR was performed using the KODFX DNA polymerase (TOYOBO), and each reaction mixture was done in a volume of 200 μl containing 72 μl H_2_O, 100 μl 2 × PCR buffer, 8 μl forward primer (10 μM), 8 μl reverse primer (10 μM), 10 μl sample DNA, and 2 μl KODFX. The parameters for amplification were as follows: initial denaturation for 2 min at 98°C; 28 cycles of denaturation at 98°C for 10 s, annealing at 55°C for 30 s, and extension at 68°C for 1 min and 40 s; and finally, an extension step at 68°C for 8 min. PCR products were visualized on agarose gel and purified using the QIA quick PCR purification kit (Qiagen), followed by quantifying on a Nanodrop 2000 (Weisburg et al., 1991).

The adaptors were ligated onto the PCR products, followed by libraries generation and sequencing using the P6-C4 chemistry on PacBio sequencing system. Sequencing was conducted on a PacBio RS II platform at TianJin Biochip Corporation, China. Raw sequences were processed through the single molecule, real-time (SMRT) Portal provided by the Pacific Biosciences RS sequencer (version 2.3.0)^1^. To ensure that the barcoded reads were correctly assigned to their original samples, a minimum barcode score of 22 was selected to achieve 99.5% accuracy. Data containing ambiguous bases were removed, primer sequences and adapters were excised from the filtered reads, and sequences outside the 10–1,490 nucleotide positions were trimmed. Analysis of 16S circular consensus sequences (CCS) was carried out using the standard tools in the Mothur package^2^, UCHIME, and Arb (Wang et al., 2007; Edgar et al., 2011; Bokulich et al., 2013).

### Operational Phylogenetic Unit Analyses

The pipeline for operational phylogenetic unit (OPU) analyses is shown in Supplementary Figure 2. Briefly, all the full-length 16S rRNA sequences were first clustered into operational taxonomic unit (OTU) with a threshold set at 98.7% identity using the USEARCH pipeline (Edgar, 2013). The most dominant sequences of each OTU was selected as the representative to be added to the LTP128 database (The All-Species Living Tree Project) (Yarza et al., 2010) and aligned using the SINA tool (SILVA Incremental Aligner) (Christian et al., 2013). The aligned sequences were inserted into the default tree using the Parsimony tool implemented in the ARB software package (Ludwig et al., 2004). The resulting insertions were manually inspected to recognize all the representative sequences closely affiliated to either type strain sequences or clearly within a genus lineage. All the sequences that remained unaffiliated were added to the SILVA REF NR database and inserted into the default tree (Christian et al., 2013). Approximately three of the closest relative sequences representing uncultured organisms were selected for each independent lineage generated by OTU representatives and inserted into the LTP128 database using the Parsimony tool. Then, a phylogenetic reconstruction was performed using the neighbor-joining algorithm and the Jukes–Cantor correction with a subset of sequences containing (i) all PacBio OTU representative sequences, (ii) the selection of the reference type strains and the SILVA REF123 recruited sequences, and (iii) the neighbor-joining supporting sequences (Munoz et al., 2014). Based on the constrained computing capability of neighbor-joining algorithm, the parsimony tree comprising of all the OTU representative sequences, reference type strains, and the selection of SILVA REF123 recruited sequences was divided into several branches accordingly. The reconstruction for each tree was performed using the 30% conservational filter to avoid phylogenetic noise.

All the OPUs were designed by the visual inspection of the final phylogenetic trees. An OPU was the smallest monophyletic group of sequences containing OTU representatives together with the closest reference sequence, including the sequence of a type strain whenever possible (Yarza et al., 2010; Christian et al., 2013). The identical or nearly identical sequences (>98.7% identity) with the type strain sequences was identified as the species with validly published names. The OPU representing an independent lineage within a clear genus was assigned as a potential new species. The OPUs representing an unclear genus, family, or higher taxon were assigned as potential higher taxa.

In general, one OPU was equal to a single bacterial species; therefore, species level phylotype (SLP) was proposed to represent all the taxa that were suggested by OPU approach, including the species with validly published names, potential new species, and potential new taxa at the levels of genus, family, order, class, or phylum.

### Compositional Analyses

The compositional analyses were performed using the R software (version 3.4.4) (Gloor et al., 2017). First, the data in the OPU table were filtered, and zero values were replaced by an estimate using the zCompositions R package. Then, the dataset was normalized by taking a centered log ratio (CLR) transformation, and the distance matrix (Aitchison distance) was calculated from the transformed data by using Euclidean distance (Martín-Fernández et al., 1998; Aitchison et al., 2000) for following principal component analysis (PCA) as well as clustering and multivariate comparison analysis. For the sample stratification, the function *hclust* with Wald.D2 method was used to cluster samples. To further determine the reliability of the generated clusters, silhouette coefficient were also calculated. Alluvial diagram was constructed by the R package ggalluvial (Smits et al., 2017) to exhibit the variation of the relative microbial abundance in different types of gut microbiota. To visualize the SLP types identified in the human fecal samples, principal coordinates analysis (PCoA) was performed by the function *cmdscale* in R package vegan to display the relationship between individual samples and the two major principal components (Cleary et al., 2012). For each pair of the SLP types generated by different methods, PCA on the two-dimensional PCoA coordinates was executed to determine the axis that explained the greatest variation among SLPs (Cleary et al., 2012). Multivariate comparison analysis (perMANOVA) was performed between each factor with the 1,235 SLPs. The variation in the Aitchison distance that can be explained by each factor was assessed using the function *adonis* in 1,000 × permutations, and the *p*-values were adjusted for multiple tests using Benjamini and Hochberg’s method (Zhernakova et al., 2016).

The CLR-transformed posterior distribution, generated by 256 Monte Carlo replicates drawn from a Dirichlet distribution, was used for quantitative analysis. The expected CLR value for each SLP was calculated, and the comparison tests between different groups were conducted using the ALDEx2 Bioconductor package. The differentially abundant SLP taxa with Benjamini–Hochberg false discovery rate (FDR) value <0.05 or |effect size| ≥1 were reported. The effect size was relatively constant, while the FDR was dependent on the sample size. Proportionality analysis was conducted using the R package propr to identify proportional abundant taxa. The highly abundant taxa with proportionality metrics ρ >0.3 were selected for illustration (Quinn et al., 2017). The 1,235 SLPs were selected to construct the network diagram. The connection between two nodes indicated that E(ρ) >0.3 or <-0.3. The size of each node was equivalent to the relative abundance of SLPs; the thickness of each edge between the two nodes was proportional to the E(ρ) (Csárdi et al., 2010). The submodule structure of the network was constructed by the fast greedy modularity optimization method and visualized using a spring-based algorithm.

### The Relative Abundance and Distribution Analysis for SLPs

Relative abundance of a given SLP in each sample was normalized as “(SLP reads/total reads) × 100 per sample.” The distribution of the entire SLPs was determined by plotting the number and relative abundance of SLP in 5% intervals (from 0 to 100% of the samples). The resulting SLP table was divided by different bacterial groups, and their proportions were analyzed separately. The Shapiro–Wilk test for normal distribution was performed directly and logarithmically.

## Results

### Hierarchic Taxonomic Composition of Gut Microbial Community

We sampled fecal specimens from 120 Chinese individuals residing in the seven historically geographic administrative regions in China, namely, the northeast (Jilin Province), east (Shandong Province), central (Henan Province), southwest (Sichuan Province), northwest (Qinghai Province), southeast (Jiangsu Province), and south (Guangdong Province) (Supplementary Figure 1). The geographic distance among the different sampling sites ranged from 259 to 3,162 km. The cohort included an equal number of healthy male and female individuals, with age ranging from 18 to 60 years old (Supplementary Table 1).

The PacBio sequencing platform rendered 1,218,217 raw 16S rRNA reads for the 120 samples. After quality filtering and chimera removal, 850,935 (69.6%) high-quality reads of 16S rRNA amplicons were obtained, with an average of 7,091.12 ± 2,751.35 reads per specimen and an average of 1,443 ± 2.68 base pairs (bp) in length per read (Supplementary Table 2). Using the USEARCH pipeline, 652,370 near full-length 16S rRNA reads were clustered into 29,787 OTUs at 98.7% identity, which was the threshold for the discrimination of bacterial species (Edgar, 2013). The most frequent representative sequences within each OTU were selected for phylogenetic inference using the LTP128 (Yarza et al., 2010) or the SILVA REF 128 NR database (Christian et al., 2013). Thereafter, 1,235 OPUs were designed based on the visual inspection of the final tree that was generated using database-based phylogenetic *de novo* tree reconstruction (Supplementary Table 3; del Mora-Ruiz et al., 2015; Vidal et al., 2015; Meng et al., 2017). An OPU was defined as the smallest monophyletic clade formed by a query sequence and a reference sequence from the databases; it included the sequence of a type strain whenever possible (del Mora-Ruiz et al., 2015; Vidal et al., 2015). Taxonomically, each OPU was equivalent to a unique bacterial species due to the relatively low divergence of the internal sequence divergence and the monophyletic structure of the clade (del Mora-Ruiz et al., 2015; Vidal et al., 2015). Therefore, we proposed to use the term species-level phylotype (SLP) to define an OPU that was similar to a species in terms of its lineage topology and identity. The SLPs were further classified in three categories, namely, classified species, potentially new species in existent genera, and potentially new lineages representing higher taxa (genus or above) (Reysenbach et al., 1994; Lozupone et al., 2012).

Rarefaction curve analysis showed high coverage but incomplete saturation (Supplementary Figures 3A,B). The average error rate generated by the PacBio sequencing platform was 0.159% (Supplementary Figure 3C), which agreed with our previous study (Meng et al., 2017). A 30% conservational filter incorporated in the LTP128 database was used to reduce the noise and to increase the accuracy of the OPU analysis (Yarza et al., 2010). The use of a conservational filter also minimized the impact of the insertions (Meng et al., 2017). Thus, we used near full-length 16S rRNA sequences and conservational filters to reconstruct the trees *de novo*, which increased the reliability of the approach due to the removal of noise and information on the sequences (Ludwig et al., 1998; Yarza et al., 2014).

**FIGURE 1:**
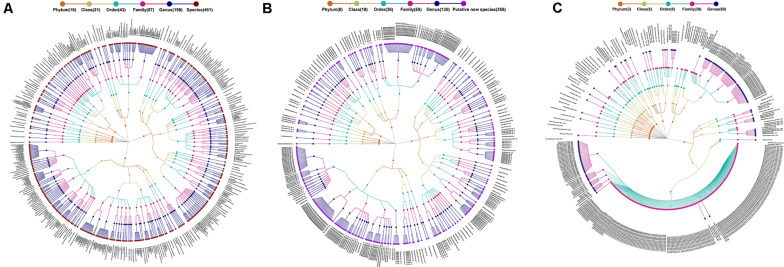
Taxonomic profiles of 1,235 species-level phylotypes (SLPs) in the gut microbial community of humans. A taxonomic tree of **(A)** 461 known species, **(B)** 368 potentially new species, and **(C)** 416 potentially higher taxa. *Each dot represents a SLP. The descending hierarchical levels are expressed from the inner to the outer rings. The total number of SPLs at different hierarchical levels is displayed in brackets.

Taxonomically, the 1,235 SLPs were comprised of 461 classified species (including four subspecies), 358 potentially new species, and 416 potentially higher taxa (Figure 1). The classified species corresponded to the SLPs where the representative 16S rRNA sequences were identical or nearly identical (>98.7% identity) with the type strain sequences of known bacterial species (Parte, 2014). The SLP representing an independent lineage within a known genus was designated as a potentially new species. The remaining 416 potentially higher taxa included 151 SLPs at the genus level, 244 SLPs at the family level, 12 SLPs at the order level, and 9 SLPs at the class or phylum level (Supplementary Figure 4; Yarza et al., 2014). Although each SLP was closely affiliated to a known genus, family, order, or higher taxa, it cannot be precisely identified by the 16S rRNA sequences alone (Rossi-Tamisier et al., 2015).

The 1,235 SLPs affiliated to 20 phyla, 36 classes, 72 orders, 121 families, and 290 genera. The 461 classified species affiliated to 198 genera, 87 families, 43 orders, 21 classes, and 10 phyla, accounting for 45.55% of the total reads (Figure 1A). The 358 potentially new species affiliated to 130 genera, 68 families, 36 orders, 18 classes, and 8 phyla, accounting for 13.71% of the reads (Figure 1B). The remaining 416 SLPs affiliated to 60 genera, 38 families, 8 orders, 5 classes, and 3 phyla, accounting for 40.47% of the total reads (Figure 1C).

### Probiotic, Commensal, and Potential Pathogenic Bacteria

To better understand the physiological roles of different gut microbial species, we categorized the 461 classified species into probiotic, commensal, and potential pathogenic bacteria, according to literature publication (Supplementary Tables 4–6). The probiotic group was composed of 12 bacterial species, dominated by the genus *Lactobacillus.* The prevalence of a single probiotic bacterial species in the cohort ranged from 0.83 to 10% (Supplementary Table 4; Kanmani et al., 2013). The probiotic bacterial species accounted for 0.03% of the total reads. The commensal bacteria group, which was comprised of non-harmful bacterial species that have not been associated with any infections in humans, was composed of 283 SLPs (61.39%), accounting for 40.82% of the total reads (McCutcheon and Moran, 2011). Six SLPs in the commensal bacteria group were shared in the gut microbiota from more than 90% of the individuals. The most prevalent commensal bacteria was *Bacteroides vulgatus*, which was present in the gut microbiota from 98.33% (118/120) of the individuals. On the other hand, the most abundant commensal bacterial species was *Prevotella copri*, which accounted for 11.71% of the total reads, followed by *B. vulgatus* (Supplementary Table 5). *P. copri* was present in the gut microbiota from 80% of individuals (Supplementary Table 5). Unexpectedly, 166 out of 461 (36.01%) of the classified bacterial species were potential pathogenic bacteria, which were previously associated with clinical infections or outbreaks (Supplementary Table 6). They accounted for 4.71% of the total reads.

### Low, Medium, and High Prevalent Bacteria Groups

**FIGURE 2:**
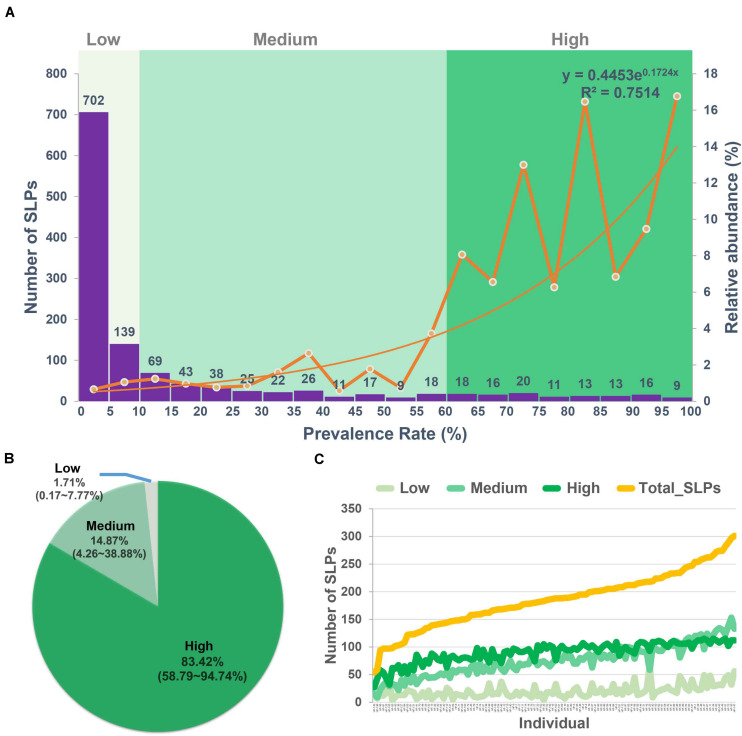
Low, medium, and high prevalent bacteria groups. **(A)** The numbers (left axis) and the relative abundance (right axis) of the species-level phylotypes (SLPs) in individuals with the 5% interval. According to the prevalence of the 1,235 SLPs, they were classified into the low (<10%), medium (10–60%), and high prevalent (>60%) bacteria groups. **(B)** The relative abundance of the low, medium, and high prevalent bacteria groups in each cohort. **(C)** The total number of SLPs and SLPs in low, medium, and high prevalent bacteria groups in each individual. The low, medium, and high prevalent bacteria groups are shown in different colors.

We classified the 1,235 SLPs into low, medium, and high prevalent bacteria groups according to their prevalence (Figure 2A and Supplementary Tables 8–10). Eight hundred forty-one out of 1,235 (68.10%) SLPs present in less than 10% of the population were classified into the low prevalent bacteria group, accounting for 1.63% of the total reads (Figures 2A,B). They were considered allochthonous, as well as temporary inhabitants of the gut microbial environment (Mcnulty et al., 2011). We classified the SLPs with a prevalence of 10% to 60% into the medium prevalent bacteria group which included 278 SLPs (Figure 2A). They accounted for 14.87% of the total reads (Figure 2B). SLPs with a prevalence greater than 60% were classified into the high prevalent bacteria group, which included 116 SLPs. They accounted for 83.51% of the total reads (Figures 2A,B).

We found that the individuals harbored 186 ± 51 SLPs on average in their gut microbiota. The mean number of SLPs in low, medium, and high prevalent bacteria groups per individual was 20 ± 11, 75 ± 29, and 90 ± 19, respectively (Figure 2C). On average, each individual harbored 90 SLPs that belong to the high prevalent bacteria group, which were selected from a pool of 116 SLPs, with a 77.58% similarity (90/116).

## Prevalence and Relative Abundance of Potential Pathogenic Species

**FIGURE 3:**
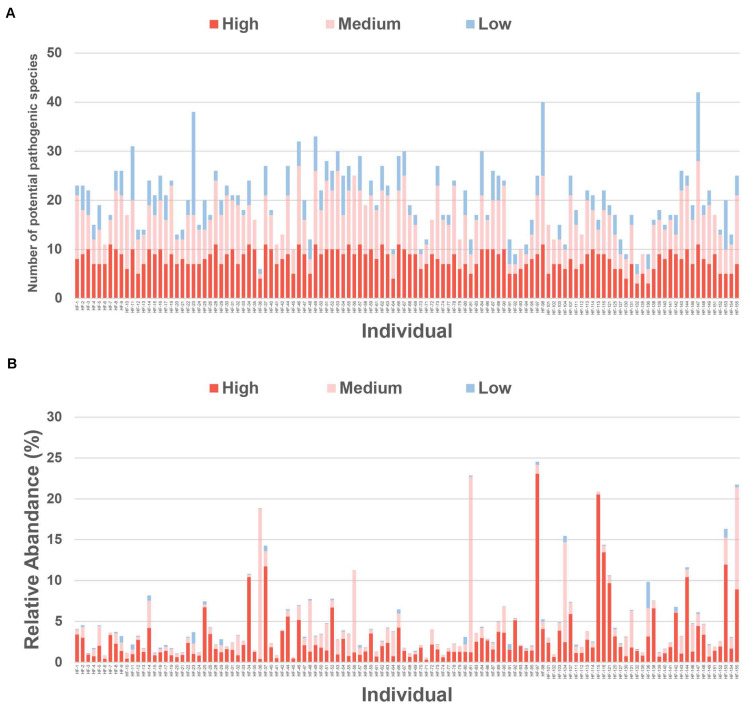
Relative abundance of potential pathogenic species in individuals. **(A)** The number of potential pathogenic species in low, medium, and high prevalent bacteria groups in individuals. **(B)** The relative abundance of potential pathogenic species in low, medium, and high prevalent bacteria groups in individuals. *The potential pathogenic species in low, medium, and high prevalent bacteria groups are shown in different colors.

The individuals had 20 ± 11 potential pathogens on average in their gut microbiota (Figure 3A). A total of 127 potential pathogenic species belonged to the low prevalent group (Figure 3A), with the remaining 39 falling into the medium or high prevalent bacteria groups (Figure 3B and Supplementary Table 7). Infections caused by the most prevalent potential pathogens detected in the gut microbiota, such as *Parabacteroides distasonis*, Bacteroides *caccae*, and *Bacteroides uniformis*, have been rarely reported (Supplementary Figure 5A). However, infections caused by the most abundant potential pathogenic species, such as *Klebsiella pneumoniae* and *B. fragilis*, have been frequently reported (Supplementary Table 6; Woo et al., 2011; Chung et al., 2012), prompting us to conclude that the overgrowth of these potential pathogenic species may result in various diseases. The species *K. pneumoniae* was present in the gut microbiota from 61.67% (74/120) of the individuals, and its relative abundance was 0.48% of the total reads, whereas its maximum relative abundance reached 22.00% of the total reads in one of the samples (Supplementary Figure 5B). Likewise, *B. fragilis* was present in the gut microbiota from 60% (72/120) of the individuals, and its maximum relative abundance reached 19.56% of the total reads in one of the samples (Supplementary Figure 5C; Zhang et al., 1999; Boleij et al., 2015). Although the prevalence of *Turicibacter sanguinis* and *Fusobacterium mortiferum* was relatively lower, their relative abundance in one of the samples accounted for 18.44 and 18.08% of the total reads, respectively (Supplementary Table 6).

### The B- and P-Type of Chinese Gut Microbial Communities

**FIGURE 4:**
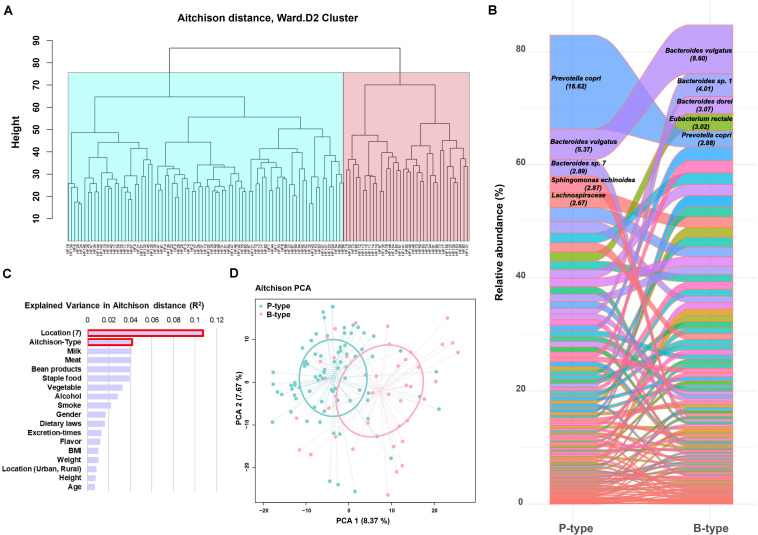
Species-level phylotype (SLP) typing of the gut microbial community in humans. **(A)** Hierarchical clustering of 120 individuals based on Aitchison distance analysis. **(B)** The relative abundance of all the 116 SLPs between the two SLP types. The abundance of the dominant species is shown. **(C)** Multivariate association analysis of the major influencing factors. **(D)** The principal component analysis (PCA) blot of proportionality data. SLPs in the three types were ordered from the highest to the lowest according to their abundance. Each SLP is represented by a single color.

Considering the relative abundance OPU table of microbiota data meets the characteristic features of a compositional data sheet, the high-throughput sequencing data were analyzed using compositional approach (R packages), which was firstly operated by normalizing the original 1,235 SLPs data with centered log ratio (CLR transformation) to construct Aitchison distance matrix (Gloor et al., 2017). The gut microbial communities of the 120 Chinese individuals were clustered into two groups, with 80 individuals in one group and 40 in another (Figure 4A). The composition of gut microflora of these two groups was analyzed by using all the 116 SLPs in the high prevalent bacterial group. Each group had a dominant species, namely, *P. copri and B*. *vulgatus*, accounting for 16.62 and 8.60% of the total reads, respectively (Figure 4B). The group dominated by *P. copri* was distributed in all the seven geographic region and was more prevalent in north China, especially in Shandong and Henan Provinces. The group dominated by *B*. *vulgatus* was more prevalent in south China (Supplementary Figure 1). Therefore the P- and B-type gut microbial communities in Chinese individuals were proposed. Multivariate comparison analysis (perMANOVA) based on Aitchison distance showed no significant association among the variance in the gender, age, height, weight, meat/vegetable consumption, and residence status (i.e., rural village or town) of these individuals. It was shown that the greatest variation among the individuals was associated with the SLP typing and geographic location (Figure 4C). The PCA of the Aitchison distance of the 1,235 SLPs revealed the most abundant species in each group, which supported our two-type gut microbiota hypothesis (Figure 4D). Furthermore, the clustering result was evaluated by calculating coefficient S(i) (silhouette) [-1 ≤ S(i) ≤ 1]. S(i) = 0.084 indicated that the classification of Chinese gut microbial community into B- and P-type was statistical reliable.

**FIGURE 5:**
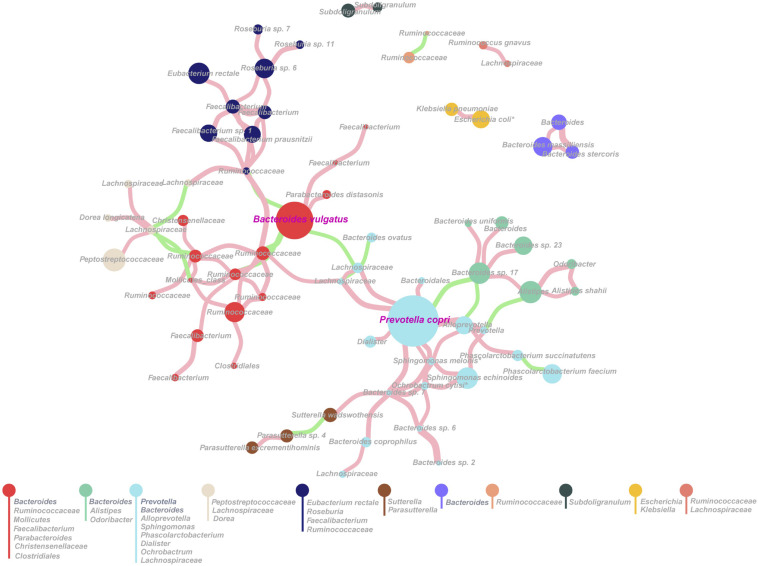
Proportional abundant species-level phylotypes (SLPs) based on proportionality analysis. Each node represents one SLP, whose abundance is directly proportional to the size of the node. Eleven modules were obtained, and each module is represented by a different color. The genus of each module is listed. An edge was drawn between SLPs if they showed a proportionality metrics (ρ) ≥0.3. A pink edge represents the proportionality between SLPs taxa, whereas a green edge indicates inverse proportionality.

We hypothesized that there was a dominant species within each type of gut microbiota, which played critical roles in the microbial community. To test the hypothesis, we performed proportionality analysis to identify proportionally abundant taxa by using R package propr (Edwards et al., 2015; Gloor et al., 2017). The highly proportional taxa among the 1,235 SLPs were calculated, which were found to form 11 co-occurrence modules (Figure 5) (the *Q*-value was 0.708, theoretically ranging from 0 to 1), indicating that the SLPs of different modules contain intimate ecological and evolutionary interactions. The major two interactions were *B. vulgatus–Ruminococcaceae* complex and *P. copri–Bacteroides–Alistipes* complex (Figure 5). We also found a negative correlation between *P. copri* and *B. vulgatus*, which further supported the two-type-gut microbiota hypothesis. The effect plot showed that the variation in most of the SLPs was greater than the difference among groups (Supplementary Figure 6), and 21 significant SLPs taxa, including *P. copri*, were detected by both Welch’s *t*-test and Wilcoxon rank sum test (Supplementary Table 14).

## Discussion

Humans are estimated to harbor 200 to more than 1,000 bacterial species in their gut intestine (Qin et al., 2010; Rajilić-Stojanović and de Vos, 2014; Costea et al., 2018), but the exact number of species in the digestive system or shared among individuals has not been determined. Studies on human gut microbial community have yielded erroneous information based on the genus level (Lozupone et al., 2012). Metagenomics has been used to study human gut microbial community in humans at the species-level, for example, Qin and Cols reported that 124 European individuals harbored approximately 1,150 bacterial species in total and that most individuals harbored approximately 160 bacterial species, of which only 63 were recognized and named previously (Qin et al., 2010). In another study, Zhernakova et al. (2016) identified 632 bacterial species in 1,135 Dutch individuals, in which 393 species had validated names. Recently, the bacterial repertoire of human gut microbiota was defined by both culture-dependent efforts and sequencing (Forster et al., 2019; Zou et al., 2019). Based on reference-free approaches, Almeida A et al. had reconstructed numerous metagenome-assembled genomes (MAGs) from human gut metagenomic datasets (Almeida et al., 2019). After sorting and removing duplicates in the datasheets of Culturable Genome Reference (CGR) (Zou et al., 2019), Human Gastrointestinal Bacteria Genome Collection (HCG) (Forster et al., 2019), and MAG (Almeida et al., 2019), 123, 286, and 99 named bacterial species were uncovered, respectively. Using metataxonomics, we found that there were at least 1,235 SLPs in the gut microbiota of 120 Chinese individuals, of which 461 species were classified with validated taxonomic names. We detected that 20 classified bacterial species detected were shared by the studies of CGR (Zou et al., 2019), HCG (Forster et al., 2019), MAG (Almeida et al., 2019), and ours (Supplementary Figure 7).

Furthermore, we found that each individual harbored 186 ± 51 SLPs on average, indicating that the metataxonomics approach used in this study, in comparison with metagenomics, could provide a more thorough species-level classification of gut microbial community than metagenomics. Metataxonomics employs OPU analysis, as well as near full-length 16S rRNA sequences, to precisely predict potentially new bacterial species or phylotypes of higher taxa by constructing the phylogenetic tree with 16S rRNA sequences of known bacterial species. It is important to emphasize that each OPU generally represents one species, as evidenced by the calculation of the intra-OPU divergence (Yarza et al., 2014). In contrast, short-gun metagenomics, especially by sequencing 16S rRNA V3–V4 region (or other variable regions), cannot precisely predict potentially new species or phylotypes given the short length of the reads.

Genus level taxonomic analysis indicated that humans share a group of bacteria in their gut intestine, which was known as the core gut microbiota (Turnbaugh et al., 2009; Martinez et al., 2013). However, using metataxonomics, we observed that no universal bacterial species was shared by the individuals included in this study, but they shared 90 ± 19 SLPs on average in a universal pool of approximately 116 bacterial species. Therefore, we must take into consideration the entire gut microbial community, not just a single species (Lozupone et al., 2012). Each individual in this cohort had a personalized gut microbiota containing a unique taxonomic composition, with a 77.8% similarity.

Interestingly, Chinese and European individuals shared a same group of high prevalent bacteria, with a certain level of similarity (Supplementary Tables 11, 12). When the high prevalent bacteria group was defined with a threshold of 60% prevalence, we found a similar number of known species in this group between Chinese and European individuals (Qin et al., 2010; Zhernakova et al., 2016), with 38, 43, and 34 in the individuals residing in China, the Netherlands, and other European countries, respectively (Qin et al., 2010; Zhernakova et al., 2016). Their similarity between Chinese individuals and individuals in the Netherlands and other European countries was 29 (67.4%) and 20 (58.8%), respectively (Supplementary Tables 11, 12). These findings indicate that, taxonomically, the high prevalent bacteria group in the gut microbiota of Chinese and European individuals had an approximate similarity of 60%. It is worth mentioning that no uniform criterion was available to classify the high prevalent bacteria group, so the different threshold values could result in various numbers of species. In previous study, the high prevalent bacteria group was considered as resident bacteria that stably colonized in human gut intestine. However, Martinson et al. (2019) reported the turnover of *Enterobacteriaceae* clones over a shot-time period, and the significant proportions of the gut microbiota were transient throughout the study period. Therefore, microbiota in human gut intestine are dynamic and potentially less stable than that were believed previously. We found that nine species in the high prevalent bacteria group were present only in Chinese individuals, while 14 species were present only in European individuals (Supplementary Table 12). By comparing the CGR (Zou et al., 2019), HCG (Forster et al., 2019), MAG (Almeida et al., 2019) and this study, we found that 12 of the 20 shared species belonged to the high prevalent bacteria group (Supplementary Table 13).

We clustered the gut microbial community of the 120 Chinese individuals into two types based on the compositional analysis of 1,235 SLPs, namely, B- and P-type (Figure 4). The B-type was dominated by *B. vulgatus* and more prevalent in the southern provinces of China. The P-type was dominated by *P. copri*, which was more prevalent in the northern provinces of China. Considering the geographical and dietary habits of the individuals residing in northern China, we hypothesized that *P. copri* may be important for the digestion of wheat and wheat-rich foods in healthy individuals. Another study showed that *Prevotella* is more common in the gut intestine of individuals following a plant-rich diet, also known as the Mediterranean diet (i.e., high levels of carbohydrates, fruits and vegetables). Recently, a study based on 26 Mongolians reported that wheat consumption as the sole carbohydrate source for an entire week suppressed the number of *Bacteroides* in the gut (Li et al., 2017). Furthermore, *P. copri* cannot produce propionate, and therefore, succinate, acetate, and formate, forming a different short-chain fatty acid composition in gut environment (Franke and Deppenmeier, 2018). Although it was hypothesized that stable foods can shape gut bacteria, the current evidence is little and more further studies are warranted.

Two or three enterotypes have been detected in the gut microbial environment, including that in Chinese individuals (Arumugam et al., 2011; Yin et al., 2017). The two genus-level enterotypes included *Bacteroides* and *Prevotella* (Liang et al., 2017; Yin et al., 2017; Costea et al., 2018), whereas Falony et al. (2016) detected *Ruminococcaceae* as well. We explored the species-level clusters of the gut microbial community, which was easily studied with sequence-based methods, such as quantitative PCR, because the representative 16S rRNA sequences of the indicator and major contributing SLPs are available. Considering that *Prevotella* and *Bacteroides* are species-rich taxa, characterized by high species and genomic diversity, future studies should focus on the bacterial species or strains in human gut microbiota, which may provide new insights on the identification of biomarker bacteria.

## Conclusion

A total of 1,235 SLPs are identified in human gut microbiota, including 774 unknown taxa. Each individual harbors 186 ± 51 SLPs on average, including 20 ± 11, 75 ± 29, and 90 ± 19 SLPs in low, medium, and high prevalent bacteria groups, respectively. There was no universal bacterial species shared among all the individuals. However, all the individuals shared a universal species pool including 116 SLPs, of which 74.38% have not been named in taxonomy. The named species in the high prevalent bacteria group are widespread in the human gut intestine. Each individual in this cohort has a personalized microbiota with a unique taxonomic composition and a 77.8% similarity. Unexpectedly, each individual harbors 20 potential pathogenic species on average. The top two most abundant and most prevalent potential pathogenic species, namely, *K. pneumoniae* and *B. fragilis*, have been reported to caused numerous infections in humans (Supplementary Figure 5; Goodwin et al., 2011; Gu et al., 2018). Through compositionally analyzing the 1,235 SLPs, the gut microbial community of the studied Chinese individuals are clustered into two types, namely P- and B-types (Figure 4), which are dominated by *P. copri* and *B. vulgatus*, respectively.

## Data Availability Statement

The datasets generated for this study can be found in the online repositories. The names of the repository/repositories and accession number(s) can be found in the article/Supplementary Material.

## Ethics Statement

The studies involving human participants were reviewed and approved by the Ethical Committee of the National Institute for Communicable Disease Control and Prevention, Chinese Center for Disease Control and Prevention, China (No. ICDC-2016007). The patients/participants provided their written informed consent to participate in this study.

## Author Contributions

JX conceived the study. JY, SL, JP, XB, XL, and DJ performed the sampling and sequencing. JY, JP, XB, RR-M, GZ, YC, WZ, and JX analyzed the data and drafted the manuscript. YW supervised the statistical analysis. All authors contributed to the article and approved the submitted version.

## Conflict of Interest

The authors declare that the research was conducted in the absence of any commercial or financial relationships that could be construed as a potential conflict of interest.

## Footnotes

1 www.pacb.com/devnet/

2 http://www.mothur.org

## Supplementary Material

FIGURE S1 Seven historically geographic administrative regions in China sampled in this study. The northeast, north, northwest, southwest, south, east and central provinces of China are shown in light blue, green, brown, violet, cyan, strawberry red and yellow, respectively. The total numbers and percentages of B- type and P- type within each sampling site are shown in a pie chart. The country name/province name is given above the pie chart (i.e., top line). n, The number of individuals sampled.

FIGURE S2 The pipeline of OPU annotation **(A)** and analysis strategy **(B)**. The OPU procedure contained three steps: Firstly, upload the representative sequences into Arb and align with LTP128 (the newest version 132); Secondly, using SINA aligner to pick SILVA_REF_NR Sequence and merge data; the last step is to build an N-J tree and manual annotation by checking the tree.

FIGURE S3 The error rate and rarefaction curve for full-length 16S rRNA sequences and SLPs. **(A)** A rarefaction curve for the reads. **(B)** A rarefaction curve for the SLPs. **(C)** The error rate for the full-length 16S rRNA sequencing using the PacBio method.

FIGURE S4 The taxonomic structure of the gut microbial community at the levels of phylum, class, order, family, and genus. Taxa rank/No, Number counted. % indicates the percentage of SLPs classified within a given taxa.

FIGURE S5 The most prevalent and most abundant potential pathogenic species in the gut microbial community of humans. **(A)** The top 15 most prevalent potential pathogenic species in individuals. **(B)** The abundance of *K*. *pneumoniae* in individuals. **(C)** The abundance of *B. fragilis* in individuals.

FIGURE S6 Effect plot **(A)** and E vs. p plot **(B)**. In both plots, each point represents an individual SLP. The blue points represent the differentially abundant SLP with a Benjamini-Hochberg false discovery rate (FDR) value less than 0.05, and the points are circled in red if their |effect size| >1 (*Prevotella copri* and *Bacteroides sp. 7*). The effect plot shows the maximum variance within the P-type or B-type vs. between group differences. The E vs. p volcano plot shows the relationship between the effect size and the FDR.

FIGURE S7 Variation of classified bacterial species detected in large scale studies. The datasheets of CGR (Zou et al., 2019), HCG (Forster et al., 2019), MAG (Almeida et al., 2019), and SLP (this study) detected 123, 286, 99, and 461 classified bacterial species, respectively.

TABLE S1 Information of 120 healthy individuals participated the study.

TABLE S2 Quality control yields of 16S rDNA sequencing by Pacbio.

TABLE S3 The OPU annotation information.

TABLE S4 List of probiotic bacterial species detected in human gut.

TABLE S5 List of commensal bacterial species detected in human gut.

TABLE S6 List of potential pathogenic species detected in human gut.

TABLE S7 The prevalence of potential pathogenic species in human gut.

TABLE S8 List of bacterial species and unclassified SLPs in the high prevalent bacterial group of the human gut.

TABLE S9 List of bacterial species and unclassified SLPs in the medium prevalent bacterial group of the human gut.

TABLE S10 List of bacterial species and unclassified SLPs in the low prevalent bacteria group of the human gut.

TABLE S11 The high prevalent bacterial species shared by individuals of Netherland and the Chinese cohort.

TABLE S12 The high prevalent bacterial species unique for the individuals of China and the Netherlands.

TABLE S13 Comparison of bacterial species among CGR, HCG, MAG, and this study.

TABLE S14 Table of 21 significant taxa detected by ALDEx2.

DATA S1 H_120_OTU.fasta.

DATA S2 OPU_procedure.

DATA S3 OPU-OTU_list.

## Abbreviations

CLR: centered log-ratio transformation
OPU: operational phylogenetic unit
OTU: operational taxonomic unit
SLP: species-level phylotype.
