# Supplementary figures and images for: Species-Level Analysis of Human Gut Microbiota With Metataxonomics
Source: Front Microbiol. 2020 Aug 26;11:2029. doi: 10.3389/fmicb.2020.02029 (PMC7479098; doi:10.3389/fmicb.2020.02029)

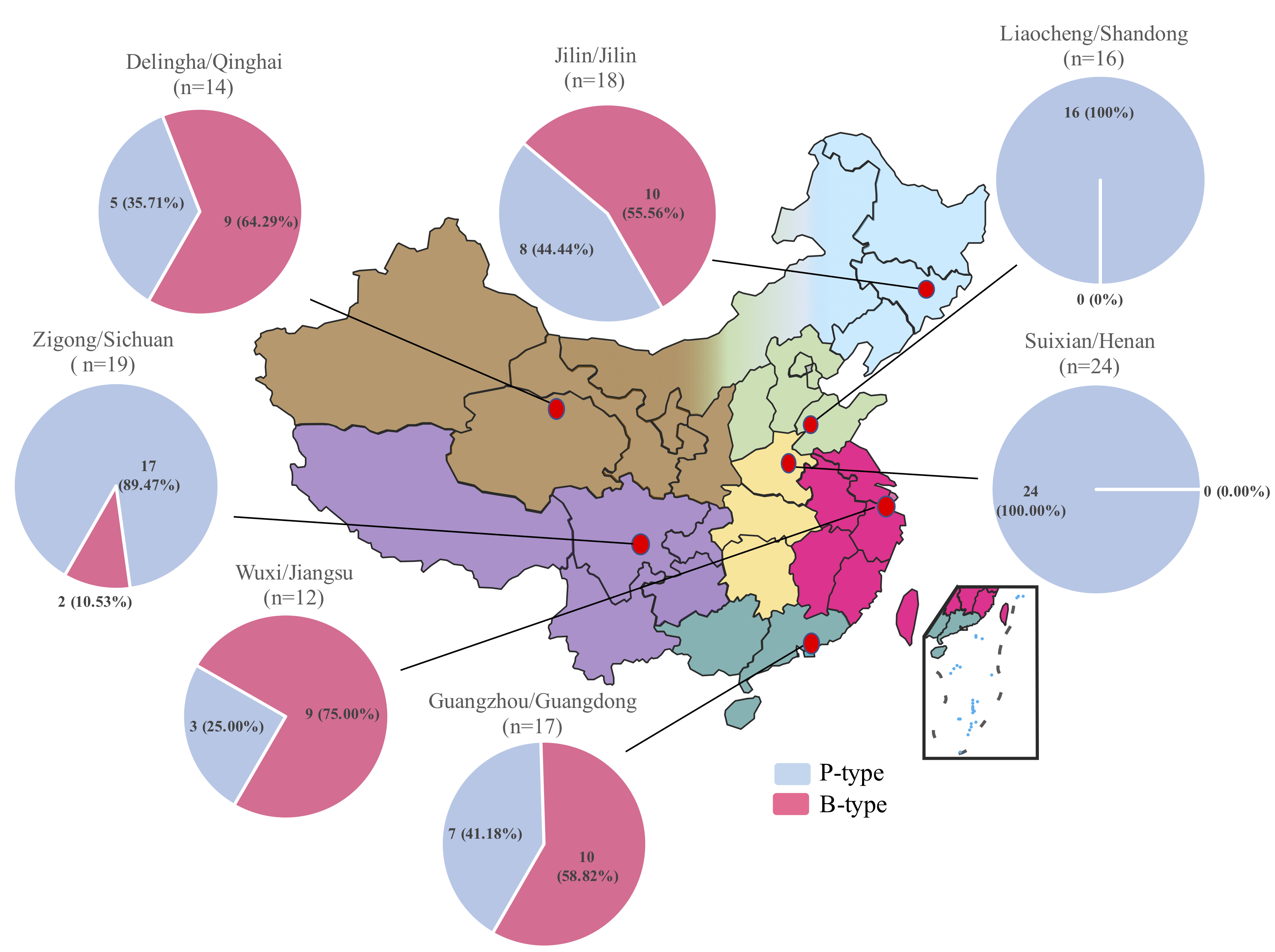

Supplement: FIGURE S1 — Seven historically geographic administrative regions in China sampled in this study. The northeast, north, northwest, southwest, south, east and central provinces of China are shown in light blue, green, brown, violet, cyan, strawberry red and yellow, respectively. The total numbers and percentages of B- type and P- type within each sampling site are shown in a pie chart. The country name/province name is given above the pie chart (i.e., top line). n, The number of individuals sampled. [file Image_1.jpg]

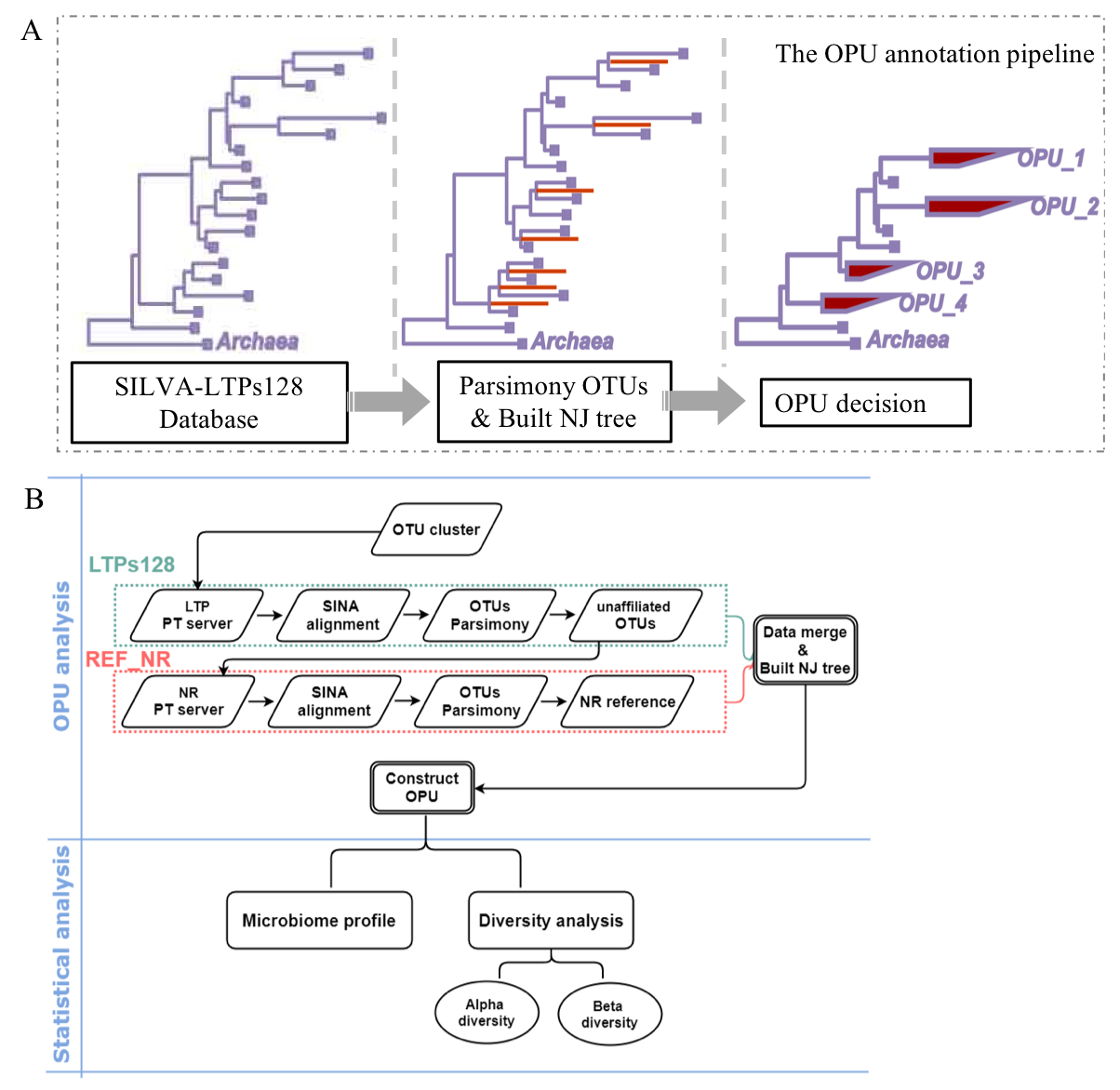

Supplement: FIGURE S2 — The pipeline of OPU annotation (A) and analysis strategy (B). The OPU procedure contained three steps: Firstly, upload the representative sequences into Arb and align with LTP128 (the newest version 132); Secondly, using SINA aligner to pick SILVA_REF_NR Sequence and merge data; the last step is to build an N-J tree and manual annotation by checking the tree. [file Image_2.jpg]

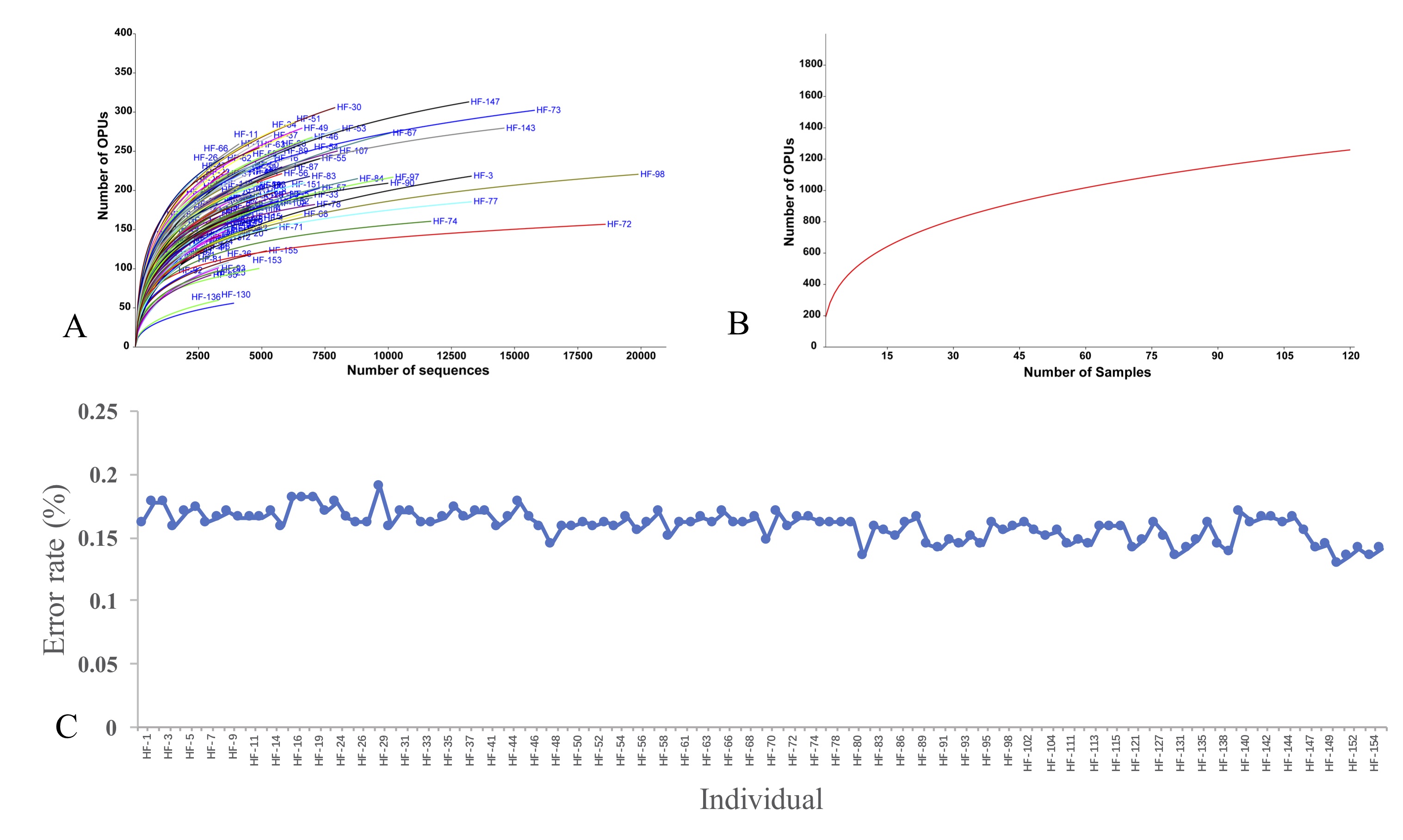

Supplement: FIGURE S3 — The error rate and rarefaction curve for full-length 16S rRNA sequences and SLPs. (A) A rarefaction curve for the reads. (B) A rarefaction curve for the SLPs. (C) The error rate for the full-length 16S rRNA sequencing using the PacBio method. [file Image_3.jpg]

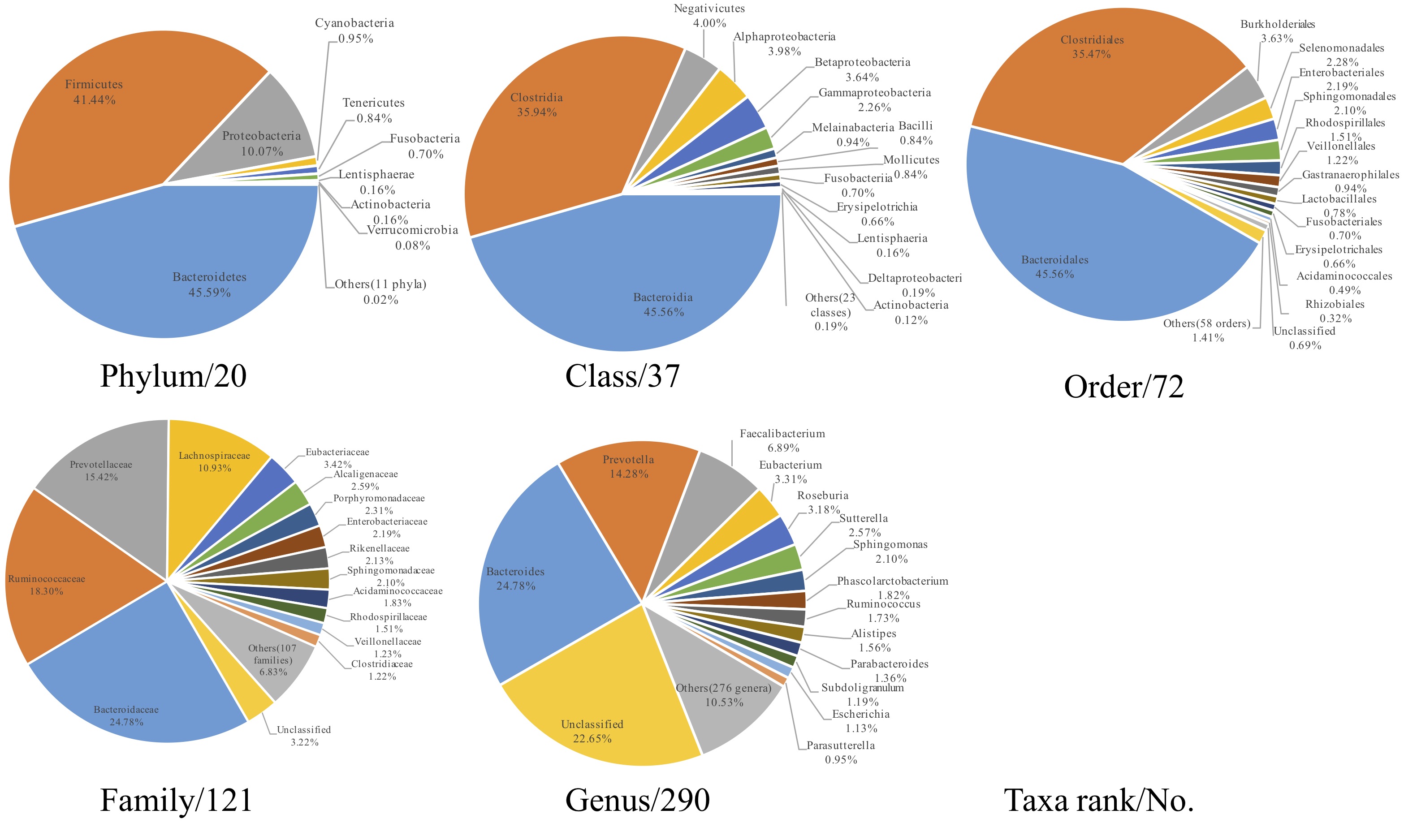

Supplement: FIGURE S4 — The taxonomic structure of the gut microbial community at the levels of phylum, class, order, family, and genus. Taxa rank/No, Number counted. % indicates the percentage of SLPs classified within a given taxa. [file Image_4.jpg]

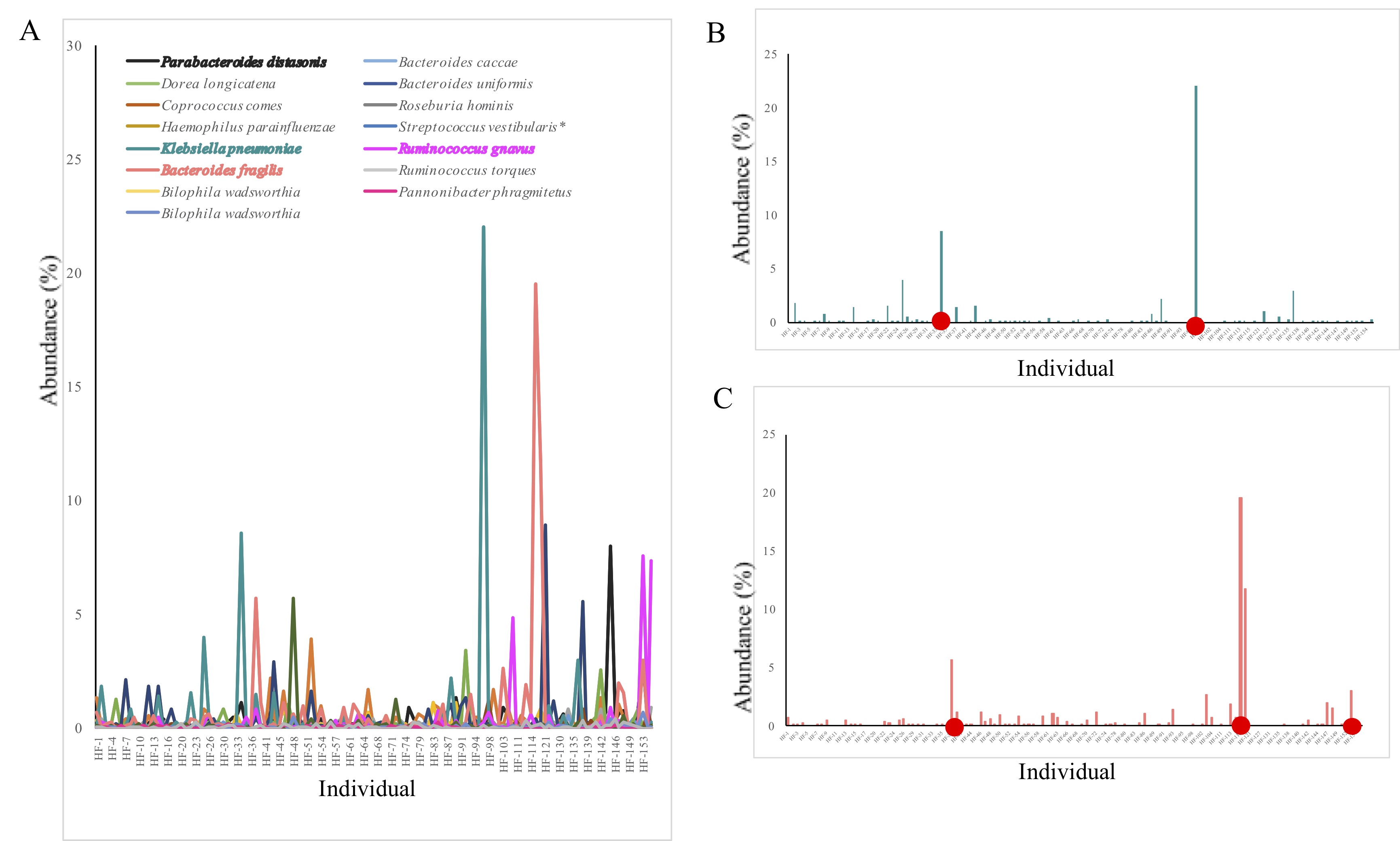

Supplement: FIGURE S5 — The most prevalent and most abundant potential pathogenic species in the gut microbial community of humans. (A) The top 15 most prevalent potential pathogenic species in individuals. (B) The abundance of K. pneumoniae in individuals. (C) The abundance of B. fragilis in individuals. [file Image_5.jpg]

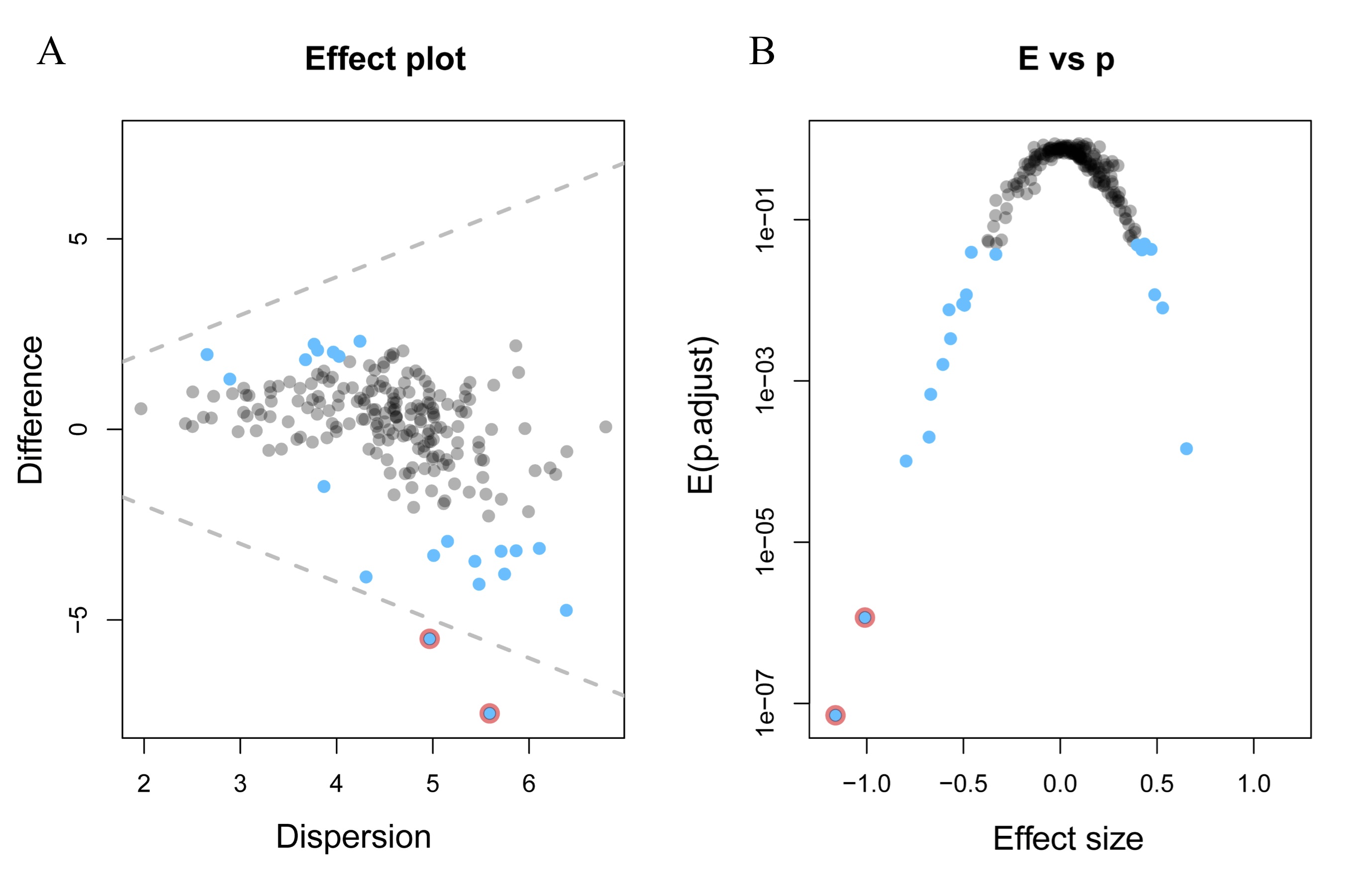

Supplement: FIGURE S6 — Effect plot (A) and E vs. p plot (B). In both plots, each point represents an individual SLP. The blue points represent the differentially abundant SLP with a Benjamini-Hochberg false discovery rate (FDR) value less than 0.05, and the points are circled in red if their |effect size| >1 (Prevotella copri and Bacteroides sp. 7). The effect plot shows the maximum variance within the P-type or B-type vs. between group differences. The E vs. p volcano plot shows the relationship between the effect size and the FDR. [file Image_6.jpg]

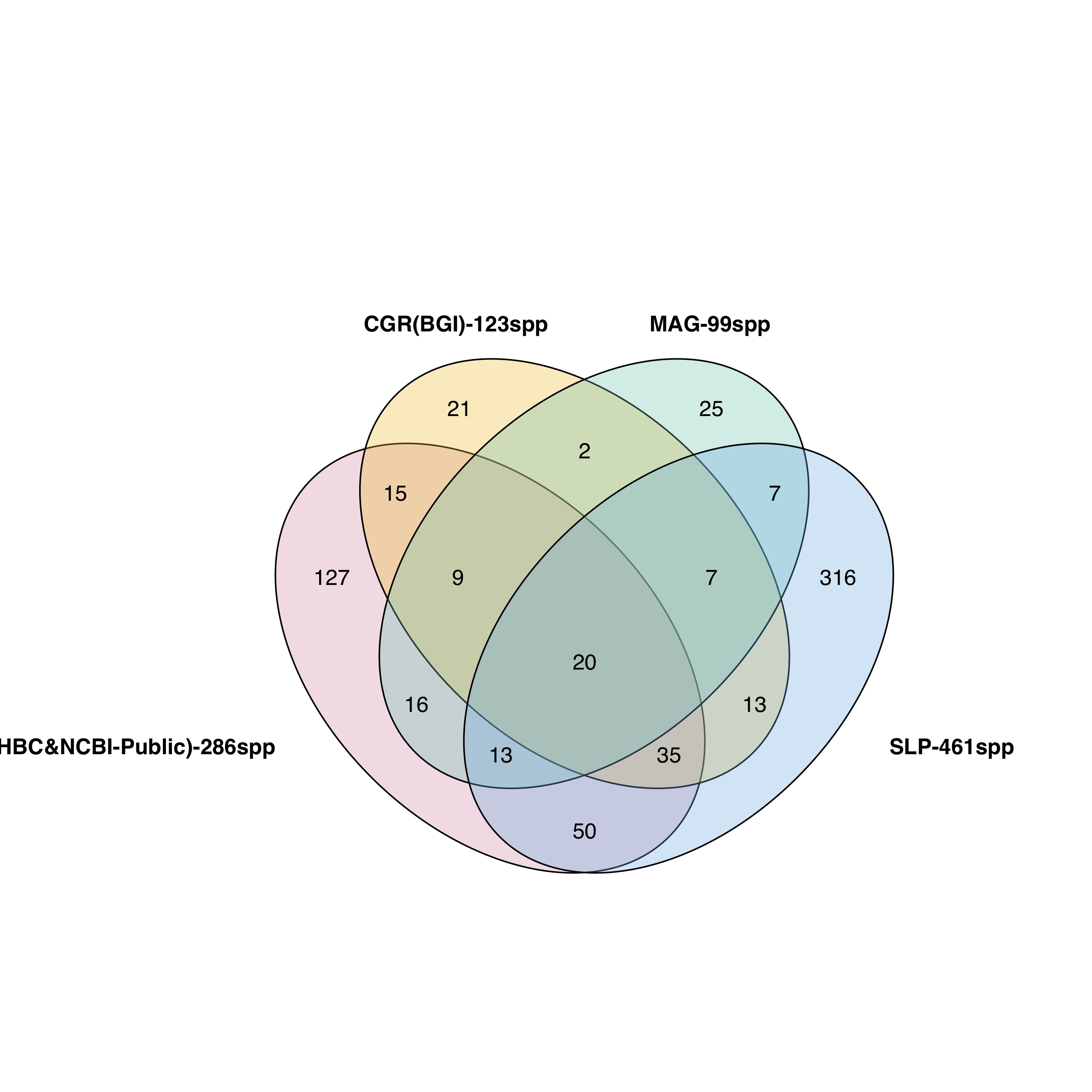

Supplement: FIGURE S7 — Variation of classified bacterial species detected in large scale studies. The datasheets of CGR (Zou et al., 2019), HCG (Forster et al., 2019), MAG (Almeida et al., 2019), and SLP (this study) detected 123, 286, 99, and 461 classified bacterial species, respectively. [file Image_7.jpg]
