# Supplementary material for: Species-Level Analysis of Human Gut Microbiota With Metataxonomics
Source: Front Microbiol. 2020 Aug 26;11:2029. doi: 10.3389/fmicb.2020.02029 (PMC7479098; doi:10.3389/fmicb.2020.02029)
Supplement: TABLE S1 — Information of 120 healthy individuals participated the study. [file Data_Sheet_1.zip › Table S11.docx]

**Table S11. The high prevalent bacterial species shared by individuals of Netherland and the Chinese cohort^1^**

| Bacterial species | Number of individuals detected positive (%) | |
| --- | --- | --- |
|  | Chinese Cohort n=120 | Netherland cohort n=1135 |
| *Bacteroides vulgatus* | 118(98.33) | 956(84.23) |
| *Faecalibacterium prausnitzii* | 116(96.67) | 1130(99.56) |
| *Parabacteroides merdae* | 112(93.337) | 805(70.93) |
| *Escherichia coli* | 111(92.50) | 701(61.76) |
| *Eubacterium rectale* | 111(92.50) | 1122(98.85) |
| *Parabacteroides distasonis* | 110(91.67) | 816(71.89) |
| *Bacteroides ovatus* | 105(87.50) | 898(79.12) |
| *Bacteroides caccae* | 104(86.67) | 789(69.52) |
| *Alistipes shahii* | 103(85.83) | 886(78.06) |
| *Dorea longicatena* | 103(85.83) | 1092(96.21) |
| *Eubacterium eligens* | 99(82.50) | 909(80.09) |
| *Roseburia intestinalis* | 99(82.50) | 856(75.42) |
| *Ruminococcus bromii* | 98(81.67) | 1026(90.40) |
| *Anaerostipes hadrus* | 96(80.00) | 1078(94.98) |
| *Prevotella copri* | 96(80.00) | 427(37.62) |
| *Bacteroides dorei* | 94(78.33) | 833(73.39) |
| *Bacteroides massilliensis* | 94(78.33) | 420(37.00) |
| *Bacteroides uniformis* | 89(74.17) | 1060(93.39) |
| *Coprococcus comes* | 89(74.17) | 1058(93.22) |
| *Roseburia hominis* | 88(73.33) | 1018(89.69) |
| *Bacteroides stercoris* | 87(72.50) | 506(44.58) |
| *Haemophilus parainfluenzae* | 87(72.50) | 264(23.26) |
| *Dorea formicigenerans* | 81(67.50) | 1099(96.83) |
| *Streptococcus vestibularis* | 75(62.50) | 404(35.59) |
| *Sutterella wadswothensis* | 75(62.50) | 361(31.81) |
| *Klebsiella pneumoniae* | 74(61.67) | 26(2.29) |
| *Ruminococcus gnavus* | 73(60.83) | 479(42.20) |
| *Bacteroides fragilis* | 72(60.00) | 394(34.71) |
| *Bacteroides thetaiotaomicron* | 72(60.00) | 788(69.43) |

1 Qin, J. *et al.* A human gut microbial gene catalogue established by metagenomic sequencing. *Nature* **464**, 59-65, doi:10.1038/nature08821 (2010).
