# Supplementary material for: Species-Level Analysis of Human Gut Microbiota With Metataxonomics
Source: Front Microbiol. 2020 Aug 26;11:2029. doi: 10.3389/fmicb.2020.02029 (PMC7479098; doi:10.3389/fmicb.2020.02029)
Supplement: TABLE S1 — Information of 120 healthy individuals participated the study. [file Data_Sheet_1.zip › Table S12.docx]

Table S12. The high prevalent bacterial species unique for the individuals of China and the Netherlands.

| Bacterial species | Present in individuals of China cohort(n=120) | Present in individuals of Netherland cohort(n=1135) |
| --- | --- | --- |
| *Roseburia faecis* | 108 | 0 |
| *Phascolarctobacterium faecium* | 107 | 0 |
| *Parasutterella excrementihominis* | 99 | 0 |
| *Lactobacillus rogosae* | 98 | 0 |
| *Ruminococcus faecis* | 92 | 0 |
| *Sphingomonas echinoides* | 88 | 0 |
| *Ochrobactrum cytisi* | 80 | 0 |
| *Fusicatenibacter saccharivorans* | 79 | 0 |
| *Sphingomonas melonis* | 72 | 0 |
| *Ruminococcus obeum* | 0 | 1132 |
| *Streptococcus salivarius* | 0 | 1082 |
| *Bifidobacterium adolescentis* | 0 | 1076 |
| *Roseburia inulinivorans* | 0 | 1045 |
| *Coprococcus catus* | 0 | 1030 |
| *Eubacterium ramulus* | 0 | 971 |
| *Alistipes putredinis* | 0 | 934 |
| *Adlercreutzia equolifaciens* | 0 | 922 |
| *Streptococcus thermophilus* | 0 | 899 |
| *Eubacterium ventriosum* | 0 | 875 |
| *Clostridium bartlettii* | 0 | 871 |
| *Akkermansia muciniphila* | 0 | 811 |
| *Gordonibacter pamelaeae* | 0 | 707 |
| *Alistipes finegoldii* | 0 | 682 |
