# Supplementary material for: Species-Level Analysis of Human Gut Microbiota With Metataxonomics
Source: Front Microbiol. 2020 Aug 26;11:2029. doi: 10.3389/fmicb.2020.02029 (PMC7479098; doi:10.3389/fmicb.2020.02029)
Supplement: TABLE S1 — Information of 120 healthy individuals participated the study. [file Data_Sheet_1.zip › Table S14.docx]

**Table S14 Table of 21 significant taxa detected by ALDEx2**

Row names indicate 21 significant SLPs taxa detected in both Welch’s t-test and Wilcoxon rank sum test.

Column names are key ALDEx2 outputs: dif.btw (median difference in clr values between groups), dif.win (median of the largest difference in clr values groups), effect (median effect size: diff.btw /max(diff.win) for all instances, overlap (proportion of effect size that overlaps 0), we.ep (expected p-value of Welch’s t test), we.eBH (expected Benjamini-Hochberg corrected p-value of Welch’s t test), wi.ep (expected p-value of Wilcoxon rank sum test), and wi.eBH (expected Benjamini-Hochberg corrected p-value of Wilcoxon rank sum test).

| **SLPs taxa** | **diff.btw** | **diff.win** | **effect** | **overlap** | **we.ep** | **we.eBH** | **wi.ep** | **wi.eBH** |
| --- | --- | --- | --- | --- | --- | --- | --- | --- |
| SLP_448 *Prevotella copri* | -7.45082 | 5.590287 | -1.16109 | 0.1292 | 6E-10 | 7.13E-08 | 1E-10 | 1.05E-08 |
| SLP_894 *Sphingomonas echinoides* | -3.19651 | 5.708959 | -0.49431 | 0.274745 | 0.000978 | 0.008676 | 0.000148 | 0.001633 |
| SLP_439 *Phascolarctobacterium faecium* | 2.314433 | 4.242272 | 0.469153 | 0.266947 | 0.009202 | 0.042805 | 4.85E-05 | 0.000786 |
| SLP_737 *Sutterella wadswothensis* | -3.12146 | 6.105787 | -0.45929 | 0.3066 | 0.007505 | 0.039376 | 0.001247 | 0.008407 |
| SLP_501 *Bacteroides coprophilus* | -3.45964 | 5.436582 | -0.56641 | 0.2492 | 0.000311 | 0.003339 | 7.44E-05 | 0.000863 |
| SLP_544 *Bacteroides ovatus* | 1.83022 | 3.67686 | 0.416619 | 0.298 | 0.011012 | 0.045996 | 0.00043 | 0.00367 |
| SLP_896 *Sphingomonas melonis* | -3.87083 | 4.305843 | -0.79602 | 0.1774 | 4.42E-06 | 0.000101 | 4.81E-08 | 2.56E-06 |
| SLP_139 *Fusicatenibacter saccharivorans* | 2.234709 | 3.766145 | 0.528936 | 0.2474 | 0.001344 | 0.008036 | 7.51E-05 | 0.000827 |
| SLP_497 *Bacteroides sp. 7* | -5.49336 | 4.966757 | -1.00908 | 0.141972 | 1.69E-08 | 1.16E-06 | 3E-10 | 3.39E-08 |
| SLP_494 *Bacteroides sp. 6* | -3.79528 | 5.744739 | -0.57424 | 0.263547 | 0.000716 | 0.007622 | 5.07E-05 | 0.000767 |
| SLP_489 *Bacteroides sp. 2* | -3.18356 | 5.864995 | -0.48465 | 0.3058 | 0.001297 | 0.011671 | 0.000573 | 0.005031 |
| SLP_532 *Bacteroides sp. 17* | 1.964805 | 2.65401 | 0.652693 | 0.230354 | 4.69E-06 | 0.000144 | 2.42E-06 | 7.72E-05 |
| SLP_140 *Fusicatenibacter sp. 1* | 2.080684 | 3.802356 | 0.488028 | 0.267746 | 0.002359 | 0.011717 | 0.00026 | 0.001962 |
| SLP_462 *Prevotella* | -4.74587 | 6.386339 | -0.66861 | 0.222356 | 3.46E-05 | 0.000681 | 3.45E-06 | 8.69E-05 |
| SLP_481 *Alloprevotella* | -4.06112 | 5.48009 | -0.677 | 0.212358 | 1.99E-05 | 0.000201 | 2.48E-06 | 6.16E-05 |
| SLP_428 *Dialister* | -1.49746 | 3.868471 | -0.33234 | 0.3466 | 0.006422 | 0.037132 | 0.007103 | 0.031812 |
| SLP_190 *Lachnospiraceae* | -2.93686 | 5.152347 | -0.50377 | 0.2934 | 0.000916 | 0.008973 | 0.000338 | 0.003213 |
| SLP_402 *Ruminococcaceae* | 1.319994 | 2.890512 | 0.397345 | 0.30114 | 0.012663 | 0.048458 | 0.001155 | 0.007503 |
| SLP_147 *Lachnospiraceae* | 2.02735 | 3.965364 | 0.435246 | 0.291542 | 0.014829 | 0.049821 | 0.000877 | 0.005424 |
| SLP_1233 *Ruminococcaceae* | 1.91788 | 4.025009 | 0.422016 | 0.296 | 0.011642 | 0.042141 | 0.0013 | 0.007779 |
| SLP_610 *Bacteroidales* | -3.30789 | 5.008078 | -0.60584 | 0.253149 | 0.0001 | 0.0016 | 3.35E-05 | 0.000524 |
